# The five-item Brief-Symptom Rating Scale as a suicide ideation screening instrument for psychiatric inpatients and community residents

**DOI:** 10.1186/1471-244X-8-53

**Published:** 2008-07-02

**Authors:** For-Wey Lung, Ming-Been Lee

**Affiliations:** 1 Department of Psychiatry, Kaohsiung Armed Forces General Hospital, Kaohsiung, Taiwan; 2 Graduate Institute of Behavior Sciences, Kaohsiung Medical University, Kaohsiung, Taiwan; 3 Department of Psychiatry, National Defense Medical Center, Taipei, Taiwan; 4 Calo Psychiatric Center, Pingtung County, Taiwan; 5 Departments of Psychiatry and Social Medicine, National Taiwan University College of Medicine, Taipei, Taiwan; 6 Director of Taiwan Suicide Prevention Center, Taipei, Taiwan

## Abstract

**Background:**

An efficient screening instrument which can be used in diverse settings to predict suicide in different populations is vital. The aim of this study was to use the five-item Brief Symptom Rating Scale (BSRS-5) as a screening instrument for the prediction of suicide ideation in psychiatric, community and general medical settings.

**Methods:**

Five hundred and one psychiatric, 1,040 community and 969 general medical participants were recruited. The community participants completed a structured telephone interview, and the other two groups completed the self-report BSRS-5 questionnaire.

**Results:**

The logistic regression analysis showed that the predictors of suicide ideation for the psychiatric group were depression, hostility and inferiority (*p *< 0.001, *p *= 0.016, *p *= 0.011), for the community group, inferiority, hostility and insomnia (*p *< 0.001, *p *< 0.001, *p *= 0.003), and for the general medical group, inferiority, hostility, depression and insomnia (*p *< 0.001, *p *= 0.001, *p *= 0.020, *p *= 0.008). The structural equation model showed the same symptom domains that predicted suicide ideation for all three groups. The receiver operating characteristic curve using the significant symptom domains from logistic regression showed that for the psychiatric group, the optimal cut-off point was 4/5 for the total of the significant dimensions (positive predictive value [PPV] = 78.01%, negative predictive value [NPV] = 79.05%), for the community group, 7/8 (PPV = 68.75%, NPV = 96.09%), and for the general medical group, 12/13 (PPV = 92.86%, NPV = 88.48%).

**Conclusion:**

The BSRS-5 is an efficient tool for the screening of suicide ideation-prone psychiatric inpatients, general medical patients, and community residents. Understanding the discriminative symptom domains for different groups and the relationship between them can help health care professionals in their preventative programs and clinical treatment.

## Background

According to the 2006 Taiwan Department of Health report, suicide was the number nine leading cause of death last year, and the number two leading cause of death among adolescents [1]. Current suicide prevention strategies involve screening of both protective and risk factors for those at high risk of suicide. Risk factors for suicide include demographic, psychiatric and familial data of suicide attempters. Protective factors include factors such as social support [2] and future orientation [3]. Further, one of the risk factors found to be strongly associated with suicide is the history of psychiatric disorders [4].

Although suicide and psychiatric disorders have a strong association, suicide can also occur in the absence of psychiatric disorders; 16.3% of the general population and 25% of general medical patients outside of psychiatric departments have had suicide ideation or attempted suicide [5]. Generally, a screening program, including those for suicide prevention, involves two stages. The first stage uses a brief and efficient screening instrument to identify those who meet the predetermined criteria, such as a certain cut-off point for such instrument. For suicide previously used instruments includes Columbia Suicide Screen [6] and Suicidal Ideation Questionnaire [7]; those screened out using such instruments will move on to the second stage of a more rigorous assessment or see a health-care professional [8].

Although suicide screening instruments have been developed in the past, the cut-off points for these instruments have only been determined for a specified age group or population. A suicide screening instrument with a determined cut-off point which can be used for all settings, including the community, general medical and psychiatric settings, is needed.

The five-item Brief Symptom Rating Scale (BSRS-5) is a screening instrument developed to screen psychiatric illnesses in non-psychiatric health settings [9]. To meet the time and resource limitations in these settings, the rating scale was shortened to five items and can be utilized in the community, as well as general medical and psychiatric settings. The BSRS-5 measures the five symptom items of anxiety, depression, hostility, interpersonal sensitivity/inferiority and insomnia. Additionally, a cut-off score of 6+ for the BSRS-5 was determined for psychiatric disorders using receiver operating characteristic (ROC) analysis [10], which is a method to measure the ability of an observer to identify a signal against a background of noise [11]. This method of analysis described a function that summarizes all possible performances of a case faced with the task of detecting a signal from noise under a curve [12].

ROC analysis is based on the signal detection theory and involves plotting the sensitivity against the false-positive fraction for every cut-off point on a measure. It is used to evaluate the discriminative performance of a screening test in distinguishing cases from non-cases, and yields a summary measure, namely the area under the curve (AUC). The AUC indicates the screening instrument's ability to discriminate between cases and non-cases, with a perfectly accurate discrimination resulting in the AUC of 1.00. The area under the ROC curve (AUC) could be regarded as the probability of correct prediction [13,14].

The purpose of this study was to assess whether the BSRS-5 can be used as an efficient screening instrument for suicide ideation in the community, as well as general medical and psychiatric settings. The discriminative validity of the five symptom domains and the relationship between these domains will be investigated. Additionally, ROC analysis was used to determine the cut-off points for each of the three groups.

## Methods

### Participants

The study was approved by the institutional review board of Kaohsiung Armed Forces General Hospital in Southern Taiwan. Three groups of participants, including those from the community, general medical and psychiatric department, were recruited. The general medical and psychiatric group participants were recruited from inpatients at a general hospital in southern Taiwan. These patients completed the self-report BSRS-5 questionnaire. Due to the practical purposes of the inability to recruit a representative sample from the community, a structured telephone interview was chosen to decrease rejection rate. The community group participants were randomly sampled from Taiwan and participated in a structured telephone interview administered by trained interviewers. The 1,040 community, 969 general medical and 501 psychiatric group participants all took part in the study voluntarily, and wrote their informed consent. Of the 1,040 participants in the community group, 497 (47.8%) were male. Of the 969 participants in the general medical group, 592 (61.1%) were male, with mean of 9.34 years of education (SD = 5.10). Within the psychiatric group, 330 (65.87%) were male, with mean of 10.84 years of education (SD = 3.42). No participant in the community and general medical group was referred who had any clinical evidence of any psychiatric disorders.

### Materials

The BSRS-5 is derived from the 50-item Brief Symptom Rating Scale [15]. The self-report survey requires respondents to answer whether they have felt tense, blue, irritated, inferior, and had trouble falling asleep in the past week. These responses are rated on a five-point Likert-type scale of 0 to 4, with 0 being not at all and 4 being extremely. The BSRS-5 has demonstrated good reliability and validity [9,10]. An additional question, "Do you have any suicide ideation", was added in the end of questionnaire.

### Statistical analysis

Multiple logistic regression analysis was used to determine which of the five symptom domains had discriminative validity for suicide ideation in the three groups. The conceptual construct between these symptom domains was further validated using structural equation analysis with the AMOS 7.0 software package (SPSS, Chicago, IL). Additionally, the ROC analysis was used to determine the cut-off points for the three groups for suicide ideation. The SPSS 15.0 software package (SPSS, Chicago, IL) was used for the multiple logistic regression and ROC analyses.

## Results

The educational levels of the general medical and psychiatric groups are shown in Table 1, and the distribution of psychiatric disorders in the psychiatric group is shown in Table 2. One hundred ninety-one (37.9%) participants had mood disorder, 137 (27.3%) had schizophrenia, 71 (14.2%) had adjustment disorder, 60 (12.0%) had substance abuse, and 42 (8.6%) had other diagnoses. Two hundred seventy-one (44.1%) of the 501 participants in the psychiatric group, 51 (4.9%) of the 1,040 community participants, and 123 (12.7%) of the 969 participants in the general medical group reported having suicide ideation in the past week.

**Table 1:** The distribution of the educational levels of the general medical (N = 969), and psychiatric (N = 501) groups

Educational level	General medical n(%)	Psychiatric n(%)
Illiterate	140 (14.45)	12 (2.40)
Elementary	188 (19.40)	69 (13.77)
High school	432 (44.58)	326 (65.07)
University or college	195 (20.12)	87 (17.37)
Graduate school	14 (1.44)	7 (1.40)

**Table 2:** The prevalence of diagnosis in the psychiatric group (N = 501)

Diagnosis	N (%)
Mood disorder	191 (37.9%)
Schizophrenia	137 (27.3%)
Adjustment disorder	71 (14.2%)
Substance abuse	60 (12.0%)
Others	42 (8.6%)

The distribution of the BSRS-5 scores among the three groups is shown in Table 3. As presented, the result of the Tukey's Post Hoc analysis shows that each item is useful for differentiating significantly between the three groups (*p *< .001). The alpha coefficient of the BSRS-5 for each group is 0.881 for the psychiatric group, 0.853 for the general medical group, and 0.786 for the community group. Additionally, the inter-item correlation for the psychiatric group is 0.60, general medical group 0.558, and the community group is 0.434.

**Table 3:** The distribution of the BSRS-5 between the community, general medical and psychiatric group

Items	Community	Medical	Psychiatric	ANOVA	Tukey Post Hoc Test
	Mean (SD)	Mean (SD)	Mean (SD)		
Anxiety	.32 (.66)	.83 (1.00)	1.86 (1.24)	F = 466.13	Community < Medical MD = -.51, *p *< .001**
				*p *< .001**	Community < Psych MD = -1.51, *p *< .001**
					Psych > Medical MD = 1.03, *p *< .001**
Anger	.48 (.77)	.74 (.98)	1.89 (1.31)	F = 362.14	Community < Medical MD = -.27, *p *< .001**
				*p *< .001**	Community < Psych MD = -1.41, *p *< .001**
					Psych > Medical MD = 1.14, *p *< .001**
Depression	.42 (.73)	.83 (1.03)	2.00 (1.38)	F = 425.41	Community < Medical MD = -.41,*p *< .001**
				*p *< .001**	Community < Psych MD = -1.59, *p *< .001**
					Psych > Medical MD = 1.17, *p *< .001**
Inferior	.35 (.73)	.48 (.89)	1.60 (1.40)	F = 305.63	Community < Medical MD = -.21, *p *< .001**
				*p *= < .001**	Community < Psych MD = -1.24, *p *< .001**
					Psych > Medical MD = 1.12, *p *< .001**
Insomnia	.46 (.86)	1.21 (1.29)	2.24 (1.33)	F = 423.71	Community < Medical MD = -.75, *p *< .001**
				*p *< .001**	Community < Psych MD = -1.78, *p *< .001**
					Psych > Medical MD = 1.03,*p *< .001**
Suicide	.07 (.38)	.22 (.66)	1.23 (1.39)	F = 393.52	Community < Medical MD = -.14, *p *< .001**
				*p *< .001**	Community < Psych MD = -1.51, *p *< .001**
					Psych > Medical MD = 1.01, *p *< .001**

*p < .05, **p < .01; MD = mean difference

The parsimonious multiple logistic regression model showed that for the psychiatric group, the symptom domains of depression, hostility and inferiority were predictive of suicide ideation (*p *< 0.001, *p *= 0.016, *p *= 0.011), with depression having the greatest predictive power (Table 4). For the community group, symptom domains of inferiority, hostility and insomnia were predictive of suicide ideation (*p *< 0.001, *p *< 0.001, *p *= 0.003) (Table 4). Inferiority had the highest predictive ability, and those who scored 1 point higher in the inferiority domain were 1.90 times more likely to have suicide ideation. For the general medical group, inferiority, hostility, depression and insomnia were predictive of suicide ideation (*p *< 0.001, *p *= 0.001, *p *= 0.020, *p *= 0.008), and inferiority had the greatest predictive power, as shown in Table 4. Gender was also analyzed as a predictive factor, but no statistically significant result was found (data not shown).

**Table 4:** The parsimonious multiple logistic regression of the predictive symptoms for suicide ideation in each group

**Psychiatric group**			
Variables	β	*p*	Exp (β)

Depression	0.79	< 0.001	2.20
Hostility	0.29	0.016	1.33
Inferiority	0.25	0.011	1.28

**Community group**			

Variables	β	*p*	Exp (β)

Inferiority	0.64	< 0.001	1.90
Hostility	0.62	< 0.001	1.85
Insomnia	0.41	0.003	1.50

**General medical group**			

Variables	β	*p*	Exp (β)

Inferiority	1.02	< 0.001	2.77
Hostility	0.46	0.001	1.59
Depression	0.35	0.020	1.41
Insomnia	0.27	0.008	1.31

**Figure 1:**
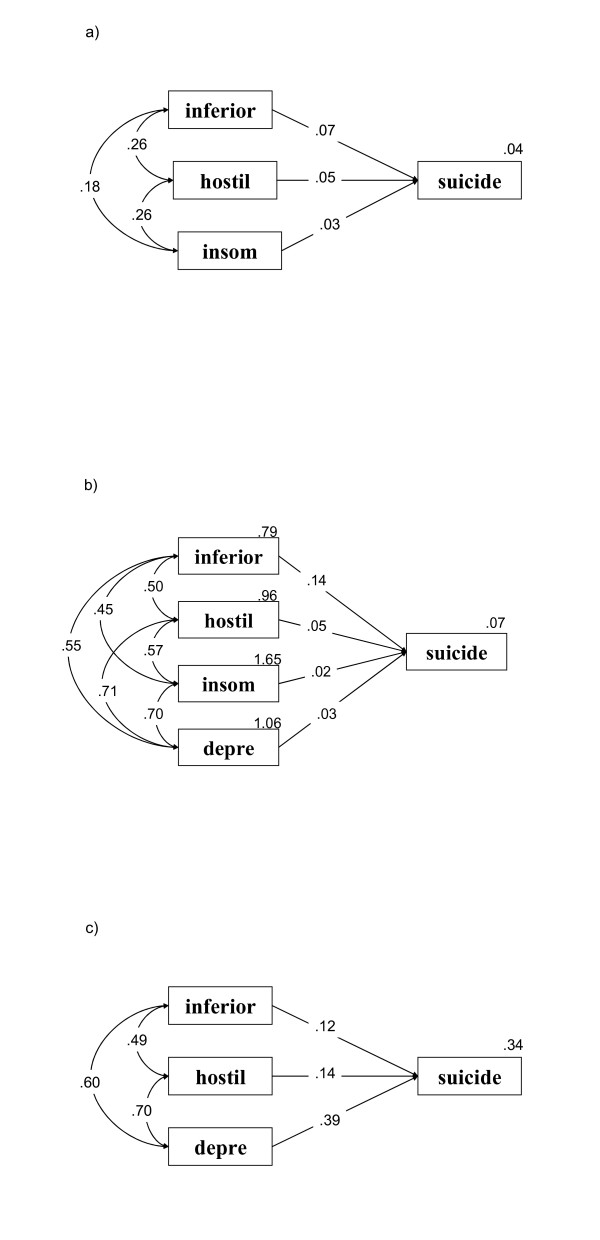
The parsimonious structural equation model of the relationship between BSRS-5 symptom domains and suicide ideation in a) the psychiatric, b) the general medical and c) the community groups inferior: inferiority; hostil: hostility; depress: depression; insom: insomnia; suicide: suicide Goodness-of-fit = 1.00 for all three groups.

The parsimonious structural equation model of the relationship between BSRS-5 symptom domains and suicide ideation in the three groups is shown in Fig. 1. All three groups analyses for the specified groups resulted in the goodness-of-fit of 1.00 (greater than 0.90), and *p *less than 0.001, therefore all models approximated the real structure.

For the psychiatric group, shown in Fig. 1a, inferiority (β = 0.12, *p *= 0.007), hostility (β = 0.14, *p *= 0.005) and depression (β = 0.39, *p *< 10^-6^) were predictive of suicide ideation. The correlation between inferiority and hostility was 0.49 (*p *< 10^-6^), between inferiority and depression, 0.60 (*p *< 10^-6^), and between hostility and depression, 0.70 (*p *< 10^-6^).

For the community group, as shown in Fig. 1b, inferiority (β = 0.23, *p *< 10^-6^), hostility (β = 0.17, *p *< 10^-6^) and insomnia (β = 0.13, *p *< 10^-4^) were predictive of suicide ideation. The correlation between inferiority and hostility was 0.46 (*p *< 10^-6^), between inferiority and insomnia, 0.29 (*p *< 10^-6^), and between hostility and insomnia, 0.39 (*p *< 10^-6^).

For the general medical group, shown in Fig. 1c, inferiority (β = 0.39, *p *< 10^-6^), hostility (β = 0.14, *p *< 10^-3^), insomnia (β = 0.07, *p *= 0.030) and depression (β = 0.10, *p *= 0.014) were predictive of suicide ideation. The correlation of inferiority with hostility, insomnia and depression was 0.57, 0.40 and 0.60, respectively (*p *< 10^-6^, *p *< 10^-6^, *p *< 10^-6^). The correlation of hostility with insomnia and depression was 0.45 and 0.70, respectively (*p *< 10^-6^, *p *< 10^-6^), and the correlation between insomnia and depression was 0.53 (*p *< 10^-6^).

**Figure 2:**
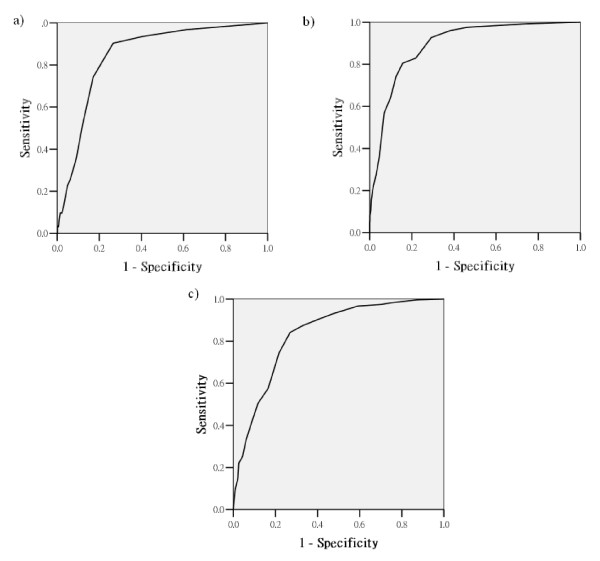
The ROC curve for the BSRS-5 result of a) the psychiatric, b) the general medical and c) the community groups.

Figure 2a shows the ROC curve for the BSRS-5 result of the psychiatric group. The AUC was 0.33, with a 95% confidence interval between 0.80 and 0.87 (*p *< 0.001). The cut-off point for the total of all five items of BSRS-5 was calculated; however, the positive predictive value (PPV), negative predictive value (NPV), sensitivity and specificity was not good (data not shown). Therefore, we used the total of the significant items to calculate the cut-off points. Table 5 shows the PPV, NPV, sensitivity and specificity of the different cut-off points. The results showed that for the psychiatric group, the optimal cut-off point was 4/5, with a PPV of 78.01% and NPV of 79.05% for the total of depression, hostility and inferiority items. Figure 2b shows the ROC curve for the BSRS-5 results of the community group. The AUC was 0.847, with a 95% confidence interval between 0.79 and 0.91 (*p *< 0.001). Table 5 shows the PPV, NPV, sensitivity and specificity of the different cut-off points using the BSRS-5 as the suicide ideation measurement. For the community group, the optimal cut-off point was 7/8, with a PPV of 68.75% and NPV of 96.09% for the total of the inferiority, hostility and insomnia items. The NPV of 96.09% implies that if the participant scored lower than 7, there was a 96.09% chance that he or she would not have suicide ideation.

**Table 5:** Validity coefficients (%) of suicide ideation in the psychiatric, community and general medical groups

	BSRS-5	PPV	NPV	Sensitivity	Specificity
Psychiatric group	4/5	78.01%	79.05%	83.76%	72.17%
Community group	7/8	68.75%	96.09%	21.57%	99.49%
General Medical group	12/13	92.86%	88.48%	10.57%	99.88%

PPV = positive predictive value; NPV = negative predictive value

Figure 2c shows the ROC curve for the BSRS-5 result of the general medical group. The AUC was 0.892, with a 95% confidence interval between 0.87 and 0.92 (*p *< 0.001). Table 5 shows the PPV, NPV, sensitivity and specificity of the different cut-off points using the BSRS-5 as the suicide ideation measurement. For the general medical group, the optimal cut-off point was 12/13, with a PPV of 92.86% and NPV of 88.48% for the total of the inferiority, hostility, depression and insomnia items. The 92.86% PPV implies that if the participant scored greater than 13, there was a 92.86% chance that he or she would have suicide ideation.

## Discussion

Different cut-off points and predictive BSRS-5 symptoms were found for suicide ideation in the psychiatric, community, and general medical group. Predictive symptom domains for the psychiatric group included depression, hostility and inferiority; inferiority, hostility and insomnia for the community group; and inferiority, hostility, depression and insomnia for the general medical group.

The predictive BSRS-5 symptom domain for suicide ideation in psychiatric inpatients was predominantly depression. This is consistent with previous studies which have found that people with psychiatric disorders, specifically borderline personality disorder and substance abuse patients, are more likely to attempt suicide when they have comorbid major depressive disorder [16,17]. Additionally, mood disorder had the highest prevalence in our psychiatric group.

For the community group, the predictive symptom domains were inferiority and hostility, and they also had a strong correlation with each other. This implies that when the individual feels inferior and is at the emotional state of anger, he or she is most likely to have thoughts of suicide. A previous study found that people without psychiatric diagnoses, but in a poor financial situation, and are males who are unemployed, which are factors that make them feel inferior, tend to be stably hopeless [18]. Furthermore, hopelessness has been determined as one of the strongest and most consistent predictors of suicidal ideation [19]. Jollant and colleagues found that suicide attempters tend to be impaired in their decision-making ability, due to emotional dysfunction and the state of anger, which is consistent with the results of this study [20].

The general medical group of inpatients exhibited the combination model of the community and psychiatric groups. This group showed that inpatients with the dominant symptoms of inferiority and hostility were most likely to form thoughts of suicide. Previous studies also found that inpatients with deliberate self-harm behavior are in a state of hopelessness, anger and global psychological distress [21].

Interestingly, anxiety and gender were not predictive factors for suicide ideation in all three models. Anxiety has been found to be a predictive factor for suicide in previous studies [22,23]. We hypothesized that anxiety was not found as a predictive factor because it is an antecedent risk factor, which appears before the feelings of depression and hopelessness [24]. When the person already has suicide ideation, only proximal factors can be found, thus anxiety was not found as a predictive factor in our models.

In all three groups, inferiority and hostility were found to be predictive symptom domains in the logistic regression and structural equation models, and they showed a strong correlation with each other. The concept of inferiority was first developed by Adler, who emphasized the interaction between the individual and society [25]. The feeling of inferiority is caused by envy or jealousy, which is caused by people's desire for social equality [26]. This feeling of inequality elicits a sense of injustice, which generally is targeted toward people of similar backgrounds [26,27]. This feeling of injustice will cause the feeling of anger, which may lead to hostility and more destructive consequences. When hostility and violence are targeted at someone else, homicide ideation will form, and when they are targeted at selves, suicide ideation will form. On the other hand, when the feeling of inferiority is directed toward someone of a superior background, this benign envy, free of hostile feelings, may be turned inward and form feelings of depression, as seen in the psychiatric group model.

Although one of the risk factors found to be strongly associated with suicide is the history of psychiatric disorders [4], less than half (45.9%) of the psychiatric inpatients reported having suicide ideation in the week before the interview. Therefore, even though psychiatric disorders are indicators for suicide, a proportion of those with psychiatric disorders never have suicide ideation or attempts. On the other hand, a proportion of people who have no history of psychiatric disorder can have suicide ideation. As found by Schairer and colleagues [28], patients with physical illness are also at greater risk for suicide, therefore, having a screening instrument which can be used in diverse settings, with a cut-off point determining suicide ideation for different populations, is vital.

Since psychiatric patients are at the highest risk of suicide, the cut off point for this group was the lowest to ensure all those at risk are not screened out. To meet the criteria for psychiatric disorder diagnosis they already have psychiatric symptoms, thus naturally scores in this psychiatric symptom screening. Additionally, the survival effect of these participants should also be taken into account. Psychiatric patients who have committed suicide would not be included in this study, thus leaves us with participants that may have less chances of committing suicide. Using the ROC analysis, we also found a cut off point of 14 being able to discriminate the psychiatric group from the other two groups. This cut off point resulted in a PPV of 78.73 and NPV of 84.37.

This study is limited by the difference in the interview method for the community and the other two groups. Although both groups used the same self-report instrument, the community group was structurally telephone-interviewed and the other two groups completed the questionnaire with the assistance of a researcher. Some participants may be more reluctant to tell the truth when interviewed on the telephone, especially regarding more personal matters such as thoughts about suicide, thus resulting in the reported significantly lower suicide ideation rate of 4.9% in the community compared to 16.7% in the general medical group. In the United States, Druss and Pincus's study found a suicide ideation or attempt rate of 16.3% in the community and 25% in the general medical group [5]. Furthermore, we did rule out whether the community or general medical group had any clinical evidence of psychiatric disorders, thus reducing the discriminative power and validity of the BSRS-5 in suicide ideation screening.

Despite these limitations, our study found that the BSRS-5 is a valid tool for the screening of those at risk of suicide in different settings. The inter-item correlation ranged from 0.43 to 0.60, which is within the range of the ideal correlation between 0.30 and 0.75. It has been stated that the ideal correlation needs to be above 0.30 to demonstrate that the items are synchronized with each other. However if the correlations between the items are too high, this implies that they have poor discriminative validity, which means the highly correlated domain could be combined or a latent variable can be found. The optimal cut-off points derived from the ROC curve for these groups were 4/5 in depression, hostility and inferiority for the psychiatric group, 7/8 in inferiority, hostility and insomnia for the community group, and 12/13 in inferiority, hostility, depression and insomnia for the general medical group. In our sample, the BSRS-5 demonstrated a PPV higher than 50% and an NPV higher than 79% (with the lowest being 79.05%). The sensitivity for the community and general medical groups was rather low (21.57% and 10.57%, respectively), reflecting the low rate of suicide ideation in these two populations; in our study, the PPV and NPV were more vital. Furthermore, the specificity and NPV for the community group were both greater than 90%, implying that those scoring lower than 8 were very unlikely to have suicide ideation. The general medical group also demonstrated good PPV and specificity greater than 90%. Lastly, the BSRS-5 only contained five items; therefore, it is efficient and easily applicable, with a determined cut-off point; it can be utilized as the first-stage screening instrument for patients in psychiatric, medical, and general community populations.

Finally, the symptom of depression was the main predictor of suicide ideation in psychiatric patients; however, inferiority and hostility were found to be the core symptom in all three groups. Our result and previous studies suggest that in different populations, their reasons for suicide ideation differ, thus having an efficient screening tool, such as BSRS-5, which can accommodate the different population is vital. Self-bolstering has been found to be an effective coping technique in reducing depression and anger for people who feel inferior [26]. Thus, it is vital for health-care professionals to focus on helping people, especially community residents, with suicide ideation to learn self-bolstering and reassurance which can promote their psychological well-being and social adaptation.

## Competing interests

The authors declare that they have no competing interests.

## Authors' contributions

All authors contributed to the design of the study. M–BL design and collected the data for the study. F–WL analyzed the data and wrote the first draft of the manuscript. Both M–BL and F–WL revised the manuscript and have approved the final manuscript.

## Pre-publication history

The pre-publication history for this paper can be accessed here:


